# A comparison of the perceived importance and performance of midwives’ roles between midwives and nurses in Korea: a cross-sectional study

**DOI:** 10.4069/kjwhn.2023.11.13

**Published:** 2023-12-28

**Authors:** Kyungwon Kim, Yunmi Kim, Jummi Park

**Affiliations:** 1 Department of Nursing, Daegu Haany University, Daegu, Korea; 2 College of Nursing, Gachon University, Incheon, Korea; 3 Department of Nursing, Namseoul University, Cheonan, Korea

**Keywords:** Nurses, Nurse midwives, Role, Perception, Work performance

## Abstract

**Purpose:**

This study aimed to identify the perceptions, importance, and performance of midwives’ roles among midwives and nurses in Korea.

**Methods:**

A descriptive correlational design was employed. Data were collected from 164 nurses and 79 midwives from April 1 to June 25, 2021. Midwives enrolled in the Korean Midwifery Association and nurses and midwives from two hospitals each Daegu and Gyeonggi Province in Korea were invited to participate. The independent t-test, chi-square test, the Welch-Aspin test, and Pearson correlation coefficient were used for analysis.

**Results:**

The midwives’ role perception score (3.47±1.46) was lower than that of nurses (3.95±0.85), and the midwives’ role performance score (2.98±0.83) was also lower than that of nurses (3.34±0.89). Significant differences were observed between midwives and nurses in their perception and performance of roles related to prenatal management, childbirth management, management of psychological changes, postpartum management, and newborn care. Higher role perception and performance among midwives were linked to the management of psychological changes and women’s health, indicating potential areas for future development.

**Conclusion:**

The study results suggest directions for developing new roles for midwives. It is necessary to find a way to expand the field of midwives in public health by benchmarking the roles of midwives in various countries.

## Introduction

Since South Korea’s total fertility rate dropped below the replacement level of two children per woman in 1985, it has fluctuated between one and just under two. In 2008, the rate was 1.19, but it reached a historic low of 0.92 in 2019, followed by 0.78 in 2022. The number of births in 2022 was particularly low at 249,100, making it a challenge to sustain numbers in the 300,000 range [1]. The domestic number of births also saw a significant decline of 37% from 434,169 in 2015 to 273,292 in 2020. Meanwhile, the number of obstetrics and gynecology hospitals and maternity hospitals—key facilities for childbirth—initially increased from 13 to 20, but then dropped to 15 by 2020. Furthermore, the number of births in maternity hospitals fell from 1,676 in 2013 to just 668 in 2020 [2]. This decline has put the profession of midwives, who focus on childbirth preparation, in jeopardy.

Midwives are specialized healthcare professionals who have been instrumental in improving public health and promoting healthy lifestyles through guidance on childbirth, as well as prenatal and neonatal care [3]. Their responsibilities encompass childbirth preparation, managing the various stages of labor (stages 1 through 4), providing postpartum care for newborns, and performing resuscitation on infants who experience asphyxiation. In the realm of postpartum care, midwives oversee breastfeeding support and care for high-risk postpartum conditions. For newborns, they conduct health assessments and offer education and counseling [4], thereby extending the scope of maternal nursing. Midwives have also been at the forefront of advocating for natural childbirth methods, minimizing unnecessary medical interventions, and empowering women to take charge of their bodies during labor, which contributes to more positive childbirth experiences [5]. However, South Korea’s record-low fertility rate has led to a situation where midwives are unable to fulfill their vital roles, putting them at risk of losing their jobs and highlighting the need to reassess their professional roles.

In various countries, in anticipation of changes in childbirth environments, guidelines for midwifery practices have been developed to explore the expanded roles of midwives across multiple aspects. In Sweden, midwives have broadened their scope of responsibilities to include tasks such as dressing cesarean section incisions, as well as duties related to family planning and counseling adolescents [6]. In the United Kingdom, midwives address health issues including domestic violence, sexual assault, mental disorders, and substance abuse. They conduct home visits, monitor the health and safety of children, assess parenting skills, and provide referrals to relevant agencies when intervention is necessary [7]. Canadian midwives offer pregnancy and parenting services, facilitate information exchange among expectant mothers, and integrate these efforts into the local community [8]. These countries emphasize the importance of expanding the scope of midwives’ roles to include the responsibilities typically associated with specialized nurses, underscoring the capability of midwives as healthcare professionals who can provide primary nursing care. By standardizing midwifery practices and extending the unique responsibilities of midwives to serve all community members, these nations are establishing a healthcare system where midwives can effectively perform specialized roles.

Anticipating changes in the role of midwives in Korean society, we examined the actual tasks performed by midwives. Our study involved analyzing job experiences in maternity hospitals [5], developing guidelines for community-based midwives [9], and researching job satisfaction among midwives in both hospitals and maternity hospitals [10,11]. Additionally, we have investigated ways to expand the role of midwives through studies on strengthening the midwifery training system [12] and broadening their scope of practice [13]. Midwives are healthcare professionals capable of independently conducting medical procedures in maternity hospitals [3], and as nurse-midwives, they also fulfill a dual role in nursing and medical procedures within obstetrics and gynecology-related healthcare settings. Consequently, by examining the perceptions of nurses and midwives, we aim to compare the perceived importance and actual frequency of midwives’ roles to identify areas that may require modification and enhancement [14].

Through this research, we aim to provide foundational data by identifying the perceived differences in the roles of midwives between nurses and midwives themselves. This data will help to broaden the scope of midwives’ roles in the community, in accordance with the responsibilities outlined in medical laws for midwifery and health guidance for pregnant women and newborns [3]. Furthermore, we plan to delineate areas where the expansion of midwives’ roles is warranted.

The purpose of this study was to identify the perception and performance of midwives’ roles among midwives and nurses, with the ultimate goal of finding strategies for expanding the roles performed by midwives. The specific objectives were as follows.

1) To understand the perception and performance of midwives’ roles among midwives and nurses.

2) To compare differences in the perception and performance of midwives’ roles among midwives.

3) To explore the relationship between the perception and performance of midwives’ roles among midwives and nurses.

## Methods

**Ethics statement:** This study was approved by the Institutional Review Board of Nam Seoul University (IRB: 202102-002). Informed consent was obtained from the participants.

### Study design

This study employed a descriptive correlational research design to assess the perception and performance of midwives’ roles among midwives and nurses. The structure and content of this study are described following research reporting guidelines, adhering to the order of the STROBE checklist (https://www.strobe-statement.org/).

### Participants

The study participants comprised midwives enrolled in the Korean Midwifery Association, as well as midwives and nurses (non-midwives) employed at two women’s specialty hospitals in Daegu and at two university hospitals that have introduced women’s healthcare services in the Gyeonggi region. Eligibility was determined based on understanding and acceptance of the study’s purpose.

The specific inclusion criteria were as follows.

1) Hold a license as a midwife or nurse (non-midwife).

2) Clearly understand the researcher’s explanations and agree to participate in the study.

The specific exclusion criteria were as follows.

1) Individuals with health conditions preventing them from reading and understanding the study questionnaire.

2) Individuals with self-reported diagnoses or experiences of depression or other mental health disorders, making their judgment unclear.

3) Individuals with a nursing license but no work experience.

The sample size for the study was calculated using G*Power version 3.15. Drawing from a prior investigation into the roles of hospital and maternity hospital midwives [15], the required sample size for two groups was determined based on an independent t-test. With a significance level set at 0.05, a medium effect size of 0.15, and a desired statistical power of 0.80, the minimum number of participants needed was 168. This study’s participants were drawn from two regions and four medical institutions, as well as from the 677 registered members of the Midwives Association. Given that a previous study on midwife core competency development, which included 681 midwives, yielded only 20% usable data [4], we aimed to include a comparable 20% of the registered midwives in the association for our sample. This approach resulted in a target group of 130 midwives and a total of 260 participants. Ultimately, 243 participants’ data were included in the final analysis.

### Study tools

#### Perception of midwives’ roles

The tool designed to assess the perception of midwives’ roles included 64 items across eight sub-domains and was originally developed by Yu [15]. We obtained permission from the developer to use the tool, which we then modified and expanded to meet the specific objectives of our study. To evaluate the content validity of the tool, we conducted a comprehensive review of both domestic and international literature on the roles of midwives, analyzed the job descriptions provided by the Korean Midwives Association, and sought content validity confirmation from three professors with expertise in women’s health nursing and 10 seasoned midwives, each with over a decade of experience. Following these steps, the tool was refined to contain 60 items within seven domains: antenatal care (18 items), intrapartum care (15 items), psychological support (three items), postpartum care (five items), neonatal care (five items), women’s health care (nine items), and management (five items). These domains were selected to gauge the perceived importance of each role. The tool employed a 5-point Likert scale, ranging from “not important” (1 point) to “very important” (5 points), allowing for total scores between 60 and 300. A higher score reflected a greater perceived importance of the role. In prior research, the tool demonstrated a Cronbach’s α value of .92 [15]. In the current study, the Cronbach’s α value reached .99, indicating excellent internal consistency. The Cronbach’s α values for the individual domains were as follows: antenatal care, .97; intrapartum care, .95; psychological support, .98; postpartum care, .98; neonatal care, .98; women’s health care, .98; and management, .95.

#### Role performance of midwifery

The tool for assessing the performance of midwives’ roles included the same items as the role perception tool developed by Yu [15]. Its content validity was established through a review of both domestic and international literature on midwives’ roles, as well as the job analysis table provided by the Korean Association of Midwives. This tool employs a Likert 5-point scale, with responses ranging from “always do” (5 points) to “never do at all” (1 point). The total score can range from 60 to 300, with higher scores indicating a greater frequency of role performance activities as perceived by the respondents. In previous research, the Cronbach’s α was reported as .92 [15], while in the current study, it was found to be .97. The Cronbach’s α values for each domain were as follows: antenatal care, .95; intrapartum care, .90; psychological support, .96; postpartum care, .94; neonatal care, .94; women’s health care, .87; and management, .87.

#### General characteristics

General characteristics included age, education level, occupation, type of workplace, workplace location, and work experience, comprising six items in total.

### Data collection

Data collection occurred between April 1 and June 25, 2021. The study was advertised on the Korean Midwifery Association’s bulletin board and nurses and midwives who were attending continuing education sessions at hospitals in Gyeonggi Province and Daegu were also invited to participate. Data collection at hospitals required obtaining permission from the institutions and making in-person visits. The researcher detailed the study’s objectives to the participants, provided them with the opportunity to review the survey, and collected signed consent forms from those who chose to participate. Completing the survey took roughly 10 to 15 minutes, and participants received small gifts (hand sanitizer and masks for coronavirus disease 2019 [COVID-19] protection) upon completion. The completed surveys were immediately collected and securely stored in a location accessible only to the researcher.

### Data analysis

The collected data were analyzed using IBM SPSS for Windows, ver. 20.0 (IBM Corp., Armonk, NY, USA). Differences in the perception of midwives’ roles between midwives and nurses, as well as differences in frequency of role performance, were analyzed using the independent sample t-test. Differences in the perception and performance of midwives’ roles among midwives were analyzed using the independent sample t-tests, and in cases where homogeneity of variance was not supported, Welch-Aspin test values were utilized. The relationship between the perception and performance of midwives’ roles among midwives and nurses was assessed using Pearson correlation coefficients.

## Results

### General characteristics of participants

Among the 243 participants, there were 79 midwives (11.7% of 677 registered midwives) and 164 nurses (non-midwives). The average age of the midwives was 46.0 years, compared to 38.2 years for the nurses. The largest age group for both the midwives and nurses was those in their 40s, representing 41.8% and 34.1% of their respective groups. Regarding educational attainment, the majority of midwives were 4-year university graduates (41.8%) and graduate school graduates (38.0%). In contrast, the nurses were predominantly 4-year university graduates (48.2%) and vocational school graduates (31.1%). The midwives were predominantly employed in general hospitals, clinics, tertiary hospitals, and private practices. The nurses most commonly worked in tertiary hospitals and general hospitals. When it came to professional experience, the largest group of midwives had 10 to less than 20 years of experience (40.5%), while the largest group of nurses had less than 10 years of experience (37.1%) (Table 1).

### Perception of midwives’ roles among midwives and nurses

For the perception of midwives’ roles midwives scored 208.74 points and nurses scored slightly higher at 235.05 points. In an analysis of average scores by area, midwives had the highest role perception score for antenatal care (3.55±1.45), followed in descending order by intrapartum care (3.54±1.25), and postpartum care (3.54±1.60). Nurses had the highest role perception score for neonatal care (4.26±1.01), followed in descending order by postpartum care (4.22±1.02), and psychological support for changes (3.80±0.98).

In antenatal care, both midwives and nurses identified referring high-risk pregnant women to a specialist (3.71±1.72 and 4.83±1.01, respectively) as the most important role of a midwife. Midwives, however, placed more emphasis on fetal heart auscultation (3.68±1.74) and assessing fetal movement (3.67±1.67), while nurses focused on educating about pain management during childbirth (4.31±0.97) and referring to a specialist upon detecting abnormal conditions in pregnant women (4.30±1.02). During intrapartum care, both groups agreed that referring to a specialist in cases of abnormal labor and dystocia (3.71±1.69 and 4.43±1.01, respectively) was an important role of midwives. Midwives gave higher priority to performing maternal cardiopulmonary resuscitation (3.80±1.59), whereas nurses emphasized the importance of family support in implementing intrapartum pain management (4.31±0.99). In providing psychological support for changes, midwives viewed offering emotional support to prevent postpartum depression (3.52±1.62) as their primary role. Nurses, in contrast, saw informing and counseling about postpartum depression reactions (4.33±0.99) as the most important. For postpartum care, education and counseling on postpartum care (3.58±1.67) were seen as essential by both midwives and nurses. Midwives added early detection, diagnosis, and treatment of postpartum complications (3.61±1.64) to their list of roles, while nurses focused on educating about prevention and self-care methods for postpartum complications (4.29±1.04) and the immediate reporting of symptoms (4.27±1.05). In neonatal care, assessing newborn health (3.65±1.75 and 4.42±1.04, respectively) and providing education and guidance on breastfeeding (3.57±1.72 and 4.25±1.09, respectively) were recognized as midwife roles by both midwives and nurses. Regarding women’s health care, counseling and referring sexual violence victims to specialized agencies (3.28±1.59 and 3.55±1.28, respectively) were considered primary midwife roles by both midwives and nurses. Midwives also included education on normal adolescent menstruation and healthy lifestyle patterns (3.29±1.59) as part of their role, while nurses considered counseling on premarital pregnancy and referrals to specialized agencies (3.57±1.25). In management, document management (3.53±1.72 and 3.81±1.21, respectively) and environmental management (3.47±1.69 and 3.80±1.20, respectively) were prioritized as primary midwife roles by both midwives and nurses (Table 2).

### Frequency of midwives’ role performance as perceived by midwives and nurses

The perceived frequency of role performance was reported to be 181.69 points by midwives and 201.10 points by nurses. When examining the average scores by area, midwives ranked their role performance frequency in the following order: neonatal care (3.29±1.38), antenatal care (3.19±1.05), intrapartum care (3.19±0.78), and postpartum care (3.19±1.25). Conversely, nurses ranked midwives' role performance frequency as follows: neonatal care (3.74±1.13), postpartum care (3.59±1.15), antenatal care (3.53±0.92), and intrapartum care (3.44±0.93).

In antenatal care, both midwives and nurses reported frequently performing roles such as assessing fetal movement, with midwives scoring 3.74±1.13 and nurses scoring 3.80±1.32. Midwives also identified fetal heart auscultation (3.58±1.75) and education on the importance of antenatal examinations and counseling (3.39±1.50) as key roles. Nurses, on the other hand, emphasized the frequent use of intrapartum pain control (3.87±1.15) and the referral of high-risk pregnant women to specialists (3.86±1.22). During intrapartum care, both groups noted a high frequency of role performance in using Doppler for fetal heart rate measurement, with midwives scoring 3.65±1.68 and nurses scoring 3.93±1.28. Midwives added intravenous injection for vascular access in emergencies (3.53±1.63) as an additional role, whereas nurses focused on supporting and encouraging mothers and families using pain control methods (3.94±1.12) and providing emergency treatment and referrals to specialists for abnormal labor and dystocia prediction (3.93±1.27). In the realm of psychological support for changes, midwives prioritized providing care to prevent postpartum depression (2.90±1.32), while nurses placed importance on informing mothers and families about postpartum psychological changes, including depression (3.45±1.23). Postpartum care engaged in education and counseling was common in both groups, with midwives scoring 3.42±1.55 and nurses scoring 3.70±1.25. Similarly, in neonatal care, both groups reported high role performance frequency in assessing newborn health, with midwives scoring 3.49±1.75 and nurses scoring 4.02±1.20. In women’s health care, midwives frequently performed roles such as early diagnosis of uterine cancer through Pap tests (2.56±1.47), education and management of elderly women (2.44±1.37), and participation in community programs or research (2.44±1.47). Nurses reported a high frequency of counseling and guidance on adolescent sexual education (2.69±1.23) and education on normal adolescent menstruation and healthy lifestyle patterns (2.67±1.22). In management tasks, both midwives and nurses indicated a high frequency of role performance in document management, with midwives scoring 3.05±1.59 and nurses 3.39±1.30, and in environmental management, with midwives scoring 2.87±1.50 and nurses 3.40±1.30 (Table 3).

### Difference in midwives’ perception and performance of midwives’ roles

The gap between midwives’ role perception and their performance was 27.05 points, with higher role perception than role performance. When examining the average scores by domain, the greatest disparity between perception and performance was observed in women’s health care, with a difference of 0.84 points. This was followed by psychological support during changes at 0.68 points, management at 0.55 points, antenatal care at 0.36 points, intrapartum care at 0.35 points, postpartum care at 0.35 points, and neonatal care at 0.24 points, as detailed in Table 4.

### Correlation between midwives’ role perception and role performance

There was a moderate positive correlation between midwives’ role perception and role performance (r=.617, *p*<.001), as well as between nurses’ role perception and role performance (r=.648, *p*<.001). Both midwives and nurses had higher scores for role perception than for role performance.

## Discussion

In terms of the importance of the seven areas of midwives’ roles, midwives prioritized antenatal care as the most crucial role, followed by intrapartum care and postpartum care. In contrast, nurses perceived neonatal care as the most important role of midwives, followed by psychological support for changes and postpartum care. There were differences in the ranking of the importance of midwives’ roles between midwives and nurses. However, regarding the frequency of role performance, both midwives and nurses reported high frequencies for neonatal care and postpartum care.

In antenatal care, nurses recognized the importance of educating expectant mothers on various topics, including antenatal examinations, breast care, nutrition, personal hygiene, and the significance of prenatal education. They also provided counseling on methods for pain control during childbirth, discussed the roles of family members, and assessed the emotional well-being and potential high-risk states of pregnant women, reporting any concerns to a specialist. This underscores the educational responsibilities of midwives, aligning with research that confirms their active engagement in these antenatal educational activities [16]. In Sweden, midwives play a comprehensive role in antenatal care, which extends to conducting ultrasounds, health assessments, and providing guidance on exercise and nutrition. They also offer parenting education, thereby enhancing women’s nursing competencies through the promotion of education, information, and overall health [6]. This broad scope of practice highlights the critical educational function of midwives in antenatal care and corroborates the findings of this study [17].

In managing labor, nurses acknowledged the significant role of midwives in various tasks, including administering intravenous injections, utilizing labor-inducing agents and uterine contraction stimulants, providing pain relief medication, monitoring fetal heart rates, promoting and facilitating intrapartum pain control educating patients about antibiotic use, and consulting with specialists in cases of abnormal childbirth and dystocia. Apart from intravenous injections, midwives are also recognized for performing house calls to adjust episiotomies for childbirth. These responsibilities, with the exception of administering analgesics, are typically included in midwifery care for natural childbirth at maternity centers [9]. Midwives advocate for hospital births to provide multidimensional care. In instances of abnormal childbirth and dystocia, they collaborate with specialists to transfer patients to the hospital, ensuring the safety of natural childbirth [16]. Although labor management is closely associated with midwifery as defined by medical laws, the ability to perform these roles is hindered by declining birth rates [1] and an increase in hospital deliveries [2]. Consequently, there is a pressing need to evaluate the role of Korean midwives in labor management and consider ways to expand their responsibilities.

Regarding psychological change support, nurses perceived midwives as playing a more crucial role than how midwives perceived it themselves in educating mothers and families about postpartum depression, as well as in counseling, care, early detection, and referral to specialists. This perception is reinforced by the fact that, in Sweden, midwives are tasked with responsibilities related to postpartum depression [6], and in the United Kingdom, they address health issues associated with mental disorders [7]. The awareness of healthcare professionals regarding the management of psychological changes could provide a foundation for broadening the scope of midwives’ roles in Korea.

In postpartum care, healthcare professionals recognize that midwives play a multifaceted role. This included family planning, providing education on postpartum care, preventing and managing postpartum complications, educating patients about the symptoms of complications, and facilitating the early detection, diagnosis, and treatment of these complications. Furthermore, midwives were also recognized as being responsible for offering family planning counseling and educating new mothers on recognizing complication symptoms and the importance of reporting them.

In neonatal care, healthcare professionals recognized midwives’ roles in assessing newborn health, educating parents on breastfeeding, providing guidance on formula feeding, advising on neonatal infection management, and counseling on infant safety. It is understood that midwives fulfill these responsibilities, with the exception of breastfeeding education and guidance. The areas of postpartum and neonatal care are encompassed within the broader scope of maternal health and well-being as defined by medical law [3]. This is true not only for midwives in Korea but also for their international counterparts who have long been engaged in these practices. For instance, in Sweden, midwives carry out these duties in maternity centers, whereas in the United Kingdom, they make home visits to assess parenting skills and offer support. In Canada, midwives play a pivotal role in fostering communication among expectant mothers within the community, and in Turkey, they leverage expertise and scientific platforms to implement these services, demonstrating a diversity of methods even within similar roles [18]. The fact that different countries adopt various strategies to enhance the effectiveness of comparable roles underscores the importance of continuous professional development for Korean midwives. This need is particularly pressing in light of the recent COVID-19 crisis and the advent of the “new normal,” as well as the challenges presented by a declining birth rate.

In the realm of women’s health care, nurses viewed midwives as pivotal in educating and guiding adolescents about normal menstrual cycles and healthy lifestyles. They are also seen as key providers of counseling and guidance on adolescent sexual education and as first responders in offering support to victims of sexual violence, including making referrals to specialized institutions. When it comes to management, nurses noted that midwives play a more significant role in environmental management than the midwives perceive themselves to have. Women’s health care is an integral part of primary healthcare. This is consistent with the duties of Swedish midwives, who provide sexual education to elementary students and adolescents, offer guidance on contraception, counsel on mental health disorders, and assist with issues of maladjustment in school life [6]. Furthermore, the roles of United Kingdom midwives in managing health issues related to domestic violence, sexual violence, mental disorders, and substance abuse are in line with the findings of this study [7,19].

Environmental management refers to healthcare professionals taking into account the hospital setting when considering patient care. Park et al. [18] suggested that the choice of expectant Korean mothers to deliver in hospitals is influenced by factors such as access to modern medical equipment and rigorous infection control, rather than the childbirth expertise of midwives. Despite this preference, satisfaction with hospital medical services remains low. Women are increasingly opting for maternity centers over hospitals to avoid issues like meconium-stained amniotic fluid, trauma, infection, and other health complications associated with hospital births. Additionally, maternity centers are favored for their more humane approach and fewer unnecessary interventions, allowing women to play a more active role in their childbirth experience [20]. This trend underscores the effective environmental management by midwives and showcases their professional expertise. Kim and Kang [21] corroborated these observations, noting that midwives place great trust in their professional organizations and public services. They value autonomy and self-regulation and have a strong sense of purpose, which contributes to higher job satisfaction compared to hospital nurses.

Midwives exhibited discrepancies between their perceived roles and their actual practices across the seven domains, with the most pronounced gaps noted in the management of women’s health and the care for psychological changes. The scope of women’s health care encompassed a range of services, including adolescent health education, sexual education, counseling for premarital pregnancy, support for single mothers, assistance for victims of sexual violence, menopause education, uterine cancer screening, management of elderly women’s health, and involvement in community programs. In the realm of psychological change care, midwives were expected to offer emotional support, counsel for postpartum depression, and facilitate the early detection and referral of postpartum depression symptoms. The management of women’s health was deemed the least important and least frequently performed task, suggesting a scarcity of opportunities, which aligns with Yu’s findings [15]. However, this contrasts with the situation in Sweden and the United Kingdom, where midwives actively engage in both women’s health management and psychological change care [6,7]. Benchmarking against these countries suggests that it is necessary to expand the roles of Korean midwives in women’s health management and psychological change care.

Lastly, the analysis of the correlations between the scores for role perception and role importance indicated that for both nurses and midwives, a higher perception of the importance of midwives’ roles was associated with higher scores for role performance. This suggests that the more midwives recognize the significance of their roles, the more frequently they perform those roles, effectively defining the scope of their responsibilities. Furthermore, since nurses perceived the roles of midwives as more important than the midwives did themselves, it is recommended that future initiatives to broaden the scope of midwives’ roles should build upon this recognition of their importance.

This study confirmed the perceived importance and performance levels of midwives’ roles as evaluated by both nurses and midwives. The higher scores assigned to midwives’ role perception and performance by nurses, as opposed to midwives themselves, suggest a need to explore new roles for midwives. This need arises particularly as they face challenges in role performance due to declining birth rates. By acknowledging the differences in role perception and performance, midwives have the opportunity to broaden their scope beyond the traditional focus on pregnant women and newborns. They can extend their services to include families and community residents, offering comprehensive health management, education, counseling, health assessments, health promotion, disease prevention, treatment, and rehabilitation within primary healthcare. Additionally, they can support psychological change management and women’s healthcare. By capitalizing on the strengths of nurse-midwives, they are well-positioned to assume diverse roles in the post-COVID-19 era in Korea.

Despite the global shortage of midwives and the clear need for midwifery training, Korea is facing challenges due to having only four institutions that offer midwife training [22]. Consequently, the training of midwives is a difficult endeavor, and even those who are trained struggle with a decreasing number of job opportunities. Although there is a general overlap between the roles of midwives and nurses worldwide, Korean midwives have traditionally concentrated exclusively on childbirth and providing health and care guidance to pregnant women and newborns. This specialization has highlighted the necessity for an evolution in the midwife’s role. Furthermore, given the significance of broadening the midwife’s role in women’s health care—especially against the backdrop of falling birth rates—there is an imperative for programs that enhance the recognition and importance of the midwife’s role.

To reinforce the essential role of midwives and broaden their responsibilities, our findings support benchmarking the roles of midwives across different countries globally. Future research that investigates the specific duties of midwives in each nation could also aid in enhancing the scope of midwifery in Korea.

## Figures and Tables

**Table 1.** General characteristics of participants (N=243)

Variable	Categories	Mean (range) or n (%)		χ^2^	*p*	Mean(range)
		Midwives (n=79)	Nurses (n=164)			
Age (year)		46.0 (26–70)	38.2 (23–65)			41.2 (23–70)
	20–29	4 (5.1)	33 (20.0)	29.95	<.001	
	30–39	18 (22.8)	52 (31.7)			
	40–49	33 (41.8)	56 (34.1)			
	50–59	12 (15.2)	22 (13.4)			
	≥60	12 (15.2)	1 (0.6)			
Education level	≥College	16 (20.3)	51 (31.1)	12.25	.007	
	University	33 (41.8)	79 (48.2)			
	≤Graduate school	30 (38.0)	34 (20.7)			
Workplace	Clinic	13 (16.5)	2 (1.2)	44.60	<.001	
	General hospital	32 (40.5)	68 (35.4)			
	Specialized general hospital	11 (13.9)	68 (41.5)			
	Private workplace	8 (10.1)	2 (1.2)			
	Other	15 (19.0)	32 (20.7)			
Region	Seoul	19 (24.1)	8 (4.9)	98.51	<.001	
	Gyeonggi	23 (29.2)	68 (41.5)			
	Chungcheong	7 (8.9)	31 (18.9)			
	Gyeongsang	26 (32.9)	56 (34.1)			
	Jeju	4 (5.1)	1 (0.6)			
Work experience (year)		21.1 (1–43)	14.0 (0.25–38)			16.2 (0.25–43)
	<10	7 (8.9)	61 (37.1)	30.13	<.001	
	10–19	32 (40.5)	54 (32.9)			
	20–29	23 (29.1)	40 (24.4)			
	≥30	17 (21.5)	9 (5.8)			

**Table 2.** Importance of midwives’ roles as perceived by midwives and nurses (N=243)

Subcategories	Importance of midwives’ roles, mean±SD		t	*p*
	Midwives	Nurses		
Antepartum care	3.55±1.45	4.02±0.86	–2.67	.009
Childbirth care	3.54±1.25	3.89±0.91	–2.34	.021
Psychological change care	3.49±1.59	4.22±0.98	–3.81	<.001
Postpartum care	3.54±1.60	4.22±1.02	–3.45	.001
Newborn care	3.53±1.63	4.26±1.01	–3.69	<.001
Women’s health care	3.23±1.50	3.49±1.16	–1.35	.179
Management	3.37±1.57	3.51±1.14	–.71	.477

**Table 3.** Midwives’ role performance frequency as perceived by midwives and nurses (N=243)

Subcategories	Midwives’ role performance frequency, mean±SD		t	*p*
	Midwives	Nurses		
Antepartum care	3.19±1.05	3.53±0.92	–2.51	.013
Childbirth care	3.19±0.78	3.41±0.91	–2.10	.037
Psychological change care	2.81±1.28	3.37±1.22	–3.32	.001
Postpartum care	3.19±1.25	3.59±1.15	–2.45	.015
Newborn care	3.29±1.38	3.74±1.13	–2.50	.014
Women’s health care	2.39±1.25	2.64±1.17	–1.55	.123
Management	2.82±1.06	3.08±1.12	–1.80	.073

**Table 4.** Differences between role perception and role performance among midwives

Subcategories	Midwives (N=79)		t	*p*
	Role perception, mean±SD	Role performance, mean±SD		
Antepartum care	3.55±1.45	3.19±1.05	3.14	.002
Childbirth care	3.54±1.25	3.19±0.78	3.18	.002
Psychological change care	3.49±1.59	2.81±1.28	3.84	<.001
Postpartum care	3.54±1.60	3.19±1.25	3.26	.002
Newborn care	3.53±1.63	3.29±1.38	2.24	.028
Women’s health care	3.23±1.50	2.39±1.25	3.40	<.001
Business management	3.37±1.57	2.82±1.06	3.27	.002
Total	3.47±1.46	2.98±0.83	3.73	<.001
